# Genetic and Functional Studies of Patients with Thyroid Dyshormonogenesis and Defects in the TSH Receptor (*TSHR*)

**DOI:** 10.3390/ijms251810032

**Published:** 2024-09-18

**Authors:** Diego Yeste, Noelia Baz-Redón, María Antolín, Elena Garcia-Arumí, Eduard Mogas, Ariadna Campos-Martorell, Núria González-Llorens, Cristina Aguilar-Riera, Laura Soler-Colomer, María Clemente, Mónica Fernández-Cancio, Núria Camats-Tarruella

**Affiliations:** 1 Growth and Development Group, Vall d’Hebron Institut de Recerca (VHIR)—Pediatric Endocrinology Section, Hospital Universitari Vall d’Hebron (HUVH), 08035 Barcelona, Spain; diego.yeste@vallhebron.cat (D.Y.); noelia.baz@vhir.org (N.B.-R.); ariadna.campos@vallhebron.cat (A.C.-M.); nuria.gonzalezllorens@vallhebron.cat (N.G.-L.); cristina.aguilar@vallhebron.cat (C.A.-R.); lsolerco.bcn.ics@gencat.cat (L.S.-C.); maria.clemente@vallhebron.cat (M.C.); monica.fernandez.cancio@vhir.org (M.F.-C.); 2 CIBERER, ISCIII, 28029 Madrid, Spain; elena.garcia@vallhebron.cat; 3 Pediatrics, Obstetrics and Gynecology and Preventive Medicine Department, Universitat Autònoma de Barcelona, 08193 Bellaterra, Spain; 4 Department of Clinical and Molecular Genetics, Vall d’Hebron University Hospital, 08035 Barcelona, Spain; maria.antolin@vallhebron.cat

**Keywords:** thyroid gland, congenital hypothyroidism, thyroid dyshormonogenesis, *TSHR*, TSH receptor, genetic variants, functional studies

## Abstract

Genetic defects in the TSH receptor (*TSHR*) can cause poor thyroid differentiation (thyroid dysgenesis) and/or thyroid malfunction (thyroid dyshormonogenesis). The phenotype spectrum is wide: from severe congenital hypothyroidism to mild hyperthyrotropinemia. Over 250 *TSHR* variants have been published, many uncharacterized in vitro. We aimed to genetically characterize patients with thyroid dyshormonogenesis with *TSHR* defects and to study in vitro the effect of the genetic variants to establish the genotype–phenotype relationship. Pediatric patients with thyroid dyshormonogenesis (160 patients, Catalan CH neonatal screening program, confirmation TSH range: 18.4–100 mIU/L), were analyzed by a high-throughput gene panel. In vitro studies measuring the TSH-dependent cAMP–response–element activation were performed. Five patients with mild or severe thyroid dyshormonogenesis presented six *TSHR* variants, two unpublished. Each variant showed a different in vitro functional profile that was totally or partially deleterious. Depending on the genotype, some of the variants showed partial deficiency in both genotypes, whereas others presented a different effect. In conclusion, the percentage of patients with thyroid dyshormonogenesis and candidate variants in *TSHR* is 3.13%. Our in vitro studies contributed to the confirmation of the pathogenicity of the variants and highlighted the importance of studying the effect of the patient’s genotype for a correct diagnostic confirmation.

## 1. Introduction

Congenital hypothyroidism (CH) is defined as a thyroid hormone deficiency that occurs at birth, which can derive from a central or primary cause. Primary or thyroid CH is the most frequent cause of CH, reported in 1:1660 to 1:4000 newborns [1,2,3,4]. It is due to defects in the gland development (agenesis or thyroid dysgenesis) or due to the alterations (partial or total blockage) of the biosynthesis of thyroid hormones (thyroid dyshormonogenesis, THD). Thyroid dysgenesis (60–70%) results from a structural defect in the thyroid gland: usually from ectopia, hypoplasia, athyreosis, or agenesis. In THD, accounting for the remaining 30–40%, a normal or enlarged eutopic thyroid gland (goiter) is usually identified [2,5].

CH patients with THD may have a genetic defect in the biosynthesis of thyroid hormones, usually transmitted in an autosomal recessive manner, whereas CH patients with thyroid dysgenesis may have defects in genes related to any step of thyroid gland development. The thyrotropin (TSH) receptor (TSHR) has a key role in the thyroid gland. It is involved in thyroid gland folliculogenesis, differentiation, and organogenesis, as well as in thyroid hormone synthesis and production.

The TSH receptor is a G-protein-coupled receptor expressed at the basolateral surface of the thyroid follicle cells. It has a classical structure: an extracellular domain connected by a hinge region to seven transmembrane domains (TMDs), with intracellular loops coupled to G proteins (Gs and Gq heterotrimers) [6]. Its large extracellular domain consists of sequential leucine-rich repeated regions (LRRs), which assemble to form a concave surface to bind the ligand TSH [6]. When TSH binds, there is a complex structural rearrangement of the receptor that is transmitted to the intracellular surface (TMD and intracellular loops). Consequently, TSHR-linked G-proteins Gsα and Gqα activate two signaling cascades: the cAMP pathway and the phosphoinositol/calcium (IP/Ca2+), respectively. The cAMP pathway is related to thyroid gland growth and differentiation, thyroid hormone secretion, and the uptake of iodide, whereas the IP/Ca2+ pathway is related to the stimulation of iodide organification [6].

The *TSHR* gene (OMIM 603372) was first described in 1995 [7]. *TSHR* genetic defects can cause poor differentiation (thyroid dysgenesis) and/or thyroid malfunction (THD). Therefore, the phenotype spectrum is wide, ranging from severe congenital hypothyroidism (CH) to mild hyperthyrotropinemia. Indeed, defects in this gene are associated with a reduced sensitivity of thyroid follicular cells to stimulation by TSH, so-called resistance to thyrotropin (RTSH) [4,6]. The degree of RTSH may depend on the type, location, and allelic dose of the *TSHR* mutations. Individuals with RTSH present with elevated serum TSH, an absence of goiter, eutopic normal-sized or hypoplastic thyroid glands, and consequent normal to low levels of thyroid hormones T3 and T4 [6,8].

More than 250 *TSHR* variants throughout its sequence have been reported. Most of the described variants are single-nucleotide variants or small indels [6,8]. Pathogenic biallelic variants are usually predicted to cause severe CH phenotype, although many of the reported variants in CH patients have not been characterized in vitro.

This study describes the clinical, biochemical, and genetic characteristics of five patients diagnosed with THD with eutopic gland and TSHR defects. The aim was to perform in vitro functional studies to confirm the pathogenicity of the variants and establish a genotype–phenotype correlation in these patients.

## 2. Results

### 2.1. Biochemical and Clinical Characteristics

We report five diagnosed patients (all males) from the Catalan CH Neonatal Screening program (N = 160) (TSH levels ≥ 10 mIU/L). Biochemical studies, gammagraphies, ultrasounds, perchlorate tests, LT4 dosage treatment, reevaluation age, and final diagnosis are summarized in Table 1. These patients were diagnosed with severe or mild THD. Clinical characteristics and anthropometric neonatal data, according to Carrascosa et al. [9], are given in Table S1.

TSH confirmation levels in the neonatal period of these patients ranged from 33.2 to 100 mIU/L. Unfortunately, we only had screening TSH levels (Table 1) of patient CH-77. Regarding neonatal clinical data to highlight, patient CH-77 was preterm (31 weeks) and CH-74 had a low birth length (SD-2.0) (Table S1). The Technetium-99m gammagraphy showed that only patient CH-71 had normal uptake whereas in three patients it was low. Four patients also presented a hypoplastic gland. All patients were treated with L-thyroxine, and three (CH-71, CH-74, CH-77) were reevaluated (ages 3–4 years). Due to high LT4 requirements, reevaluation was not carried out for patients CH-72 and CH-75. At reevaluation, iodide gammagraphies showed normal uptake in two (CH-71 and CH-77) and no uptake in CH-74. The perchlorate discharge test in CH-71, CH-74 and CH-77 was negative (Table 1). The LT4 dose (mcg/kg/day) ranged from 1.8 to 4.4 mcg/kg/day. All patients’ final diagnosis was of permanent CH.

Regarding family history, patient CH-72’s mother had gestational CH, CH-75’s father an untreated hyperthyrotropinemia, and CH-77’s father was an untreated carrier.

### 2.2. Genetics

**Figure 1:**
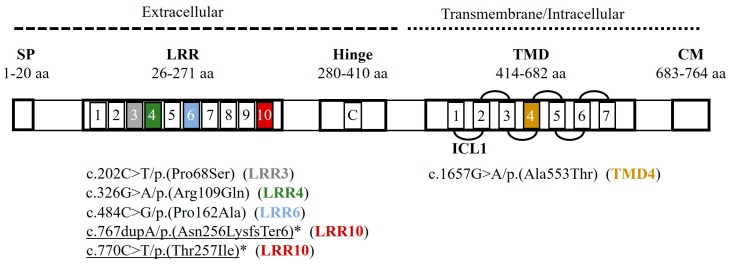
Genetic variants reported in this study in the peptide structure of the TSH receptor (TSHR, NP_000360.2). Five *TSHR* variants were located in the extracellular loops (LRR1-LRR10) and one in the membrane (TMD4). Colored squares identify the localization of each detected variant. SP: signal peptide; LRR: Leucine Reach Repeats (10 loops); C: C peptide; TMD: transmembrane domain; CM: Cytoplasmic Motifs; L: loop; ICL: intracellular. Underlined: novel variants; *: non-reported in vitro studies. Data from [6]; TSH Receptor Mutation Database (accessed on 2 August 2023, https://www.tsh-receptor-mutation-database.org/); UniProt (AC P16473; accessed on 2 August 2023, https://www.uniprot.org/uniprotkb/P16473/entry).

Six different variants were detected in these five patients (3.13% of the DHT cohort), two of which had not been previously published: c.767dupA/p.(Asn256LysfsTer6) and c.770C>T/p.(Thr257Ile), both in patient CH-71 (Figure 1, Table 2). The patients had variable genotypes: three were homozygous (CH-72, CH-74, and CH-75), one cis-heterozygous (two variants in the same allele) (CH-71), and one heterozygous (CH-77) (Table 1). All variants were single-nucleotide variations: five were missense and one was a duplication producing a frameshift [c.767dupA/p.(Asn256LysfsTer6)] (CH-71).

All tested parents carried the variants. CH-71’s father carried both *TSHR* variants (c.770C>T+c.767dupA) in cis. CH-72’s mother was a carrier (c.484C>G/p.(Pro162Ala) and had gestational CH. CH-75’s father, who had hyperthyrotropinemia, carried the *TSHR* variant c.326G>A/p.(Arg109Gln).

### 2.3. In Silico Studies

**Figure 2:**
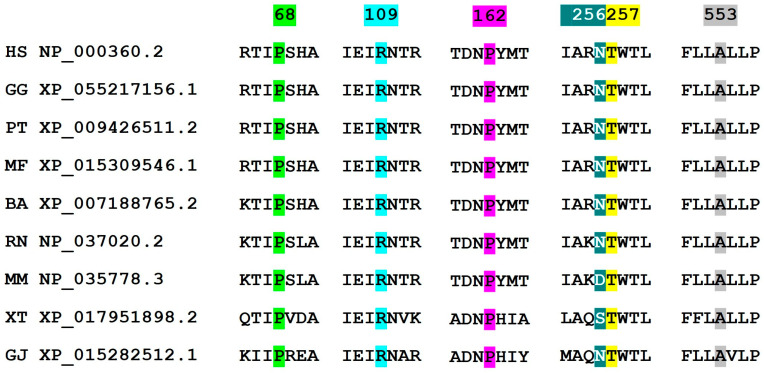
Multiple sequence alignment of the detected *TSHR* variants with eight species (Clustal Omega, EMBL-EBI). All affected amino acids except Asn256 are well conserved within species. Abbreviation (species/subspecies; name; reference sequence): HS (human; *Homo sapiens*; NP_000360.2), GG (gorilla; *Gorilla gorilla*; XP_055217156.1), PT (common chimpanzee; *Pan troglodytes*; XP_009426511.2), MF (crab-eating macaque; *Macaca fascicularis*; XP_015309546.1), BA (minke whale; *Balaenoptera acutorostrata*; XP_007188765.2), RN (brown rat; *Rattus norvegicus*; NP_037020.2), MM (house mouse; *Mus musculus*, NP_035778.3), XT (Western clawed frog; *Xenopus tropicalis*; XP_017951898.2), and GJ (Japanese gecko; *Gekko japonicus*; XP_015282512.1). Numbers indicate the amino acid position in the peptide sequence.

Two genetic variants were classified as pathogenic, and four as VUS, following ACMG guidelines (2021) [10]. All variants were exonic and located along the TSHR peptide sequence (Figure 1). Five genetic variants were located in the extracellular loops (LRR1, LRR3, LRR4, LRR6, and LRR10), and one [c.1657G>A/p.(Ala553Thr)] inside the membrane (TMD4) (Figure 1). The peptide alignment to study the conservation of affected amino acids showed that Asn256 (corresponding to nucleotide position c.767) presented two changes within primates (mouse and amphibian Xenopus). The other amino acids were well conserved within species (0 changes) (Clustal Omega, Figure 2, and Table 2).

The genetic variant affecting amino acid 256 consisted of a duplication predicted to produce a Lys256 change followed by a frameshift. In addition, all variants had a General MutPred Score over 0.5 [MutPred2, 0 (benign) − 1 (pathogenic)], indicating a tendency to pathogenicity (17) (Table 2). Furthermore, a simulation analysis (I-Mutant, pH = 7, 37 °C) of our protein sequence stability also indicated that all missense variants produced a loss in protein sequence stability (Table 2). Finally, the SpliceAI webtool showed that none of the variants were predicted to cause a splicing defect.

**Table 2:** *TSHR* genetic variants in this study.

Gene Change (NM_000369.5)	Amino Acid Change (NP_000360.2)	Gene Location/Receptor Location ^1^	Mutation Type	ACMG Classification (2021) ^2^	ClinVar (June 2023) ^2^	Conservation (ClustalO, August 2023) ^3^	MutPred2 Analysis (August 2023) ^4^	Protein sequence Stability (I-Mutant) ^6^	HGMD Classification (August 2023) ^7^	Previously Reported (First Report)/Reported Functional Studies
c.202C>T	p.(Pro68Ser)	exon 2/ECL	SNV/miss	VUS	Conflicting	+++	0.672	Decreased (−1.77)	DM	[8]/[8,11]
c.326G>A	p.(Arg109Gln)	exon 4/ECL	SNV/miss	P	P	+++	0.536	Decreased (−1.28)	DM	[12]/[12,13]
c.484C>G	p.(Pro162Ala)	exon 6/ECL	SNV/miss	VUS	P/LP	+++	0.562	Decreased (−1.94)	DM	[7]/[7,14,15]
c.767dupA	p.(Asn256LysfsTer6)	exon 9 /ECL	SNV/frameshift	P	-	+	0.505 ^5^	-	-	No/-
c.770C>T	p.(Thr257Ile)	exon 9/ECL	SNV/miss	VUS	-	+++	0.690	Decreased (−0.82)	-	No/-
c.1657G>A	p.(Ala553Thr)	exon 10/TMD	SNV/miss	VUS	Conflicting	+++	0.759	Decreased (−4.08)	DM	[16]/[16]

ECL: extracellular domain; TMD: transmembrane domain; SNV: single nucleotide variant; miss: missense; VUS: variant of uncertain significance; LP: likely pathogenic; P: pathogenic; DM: damaging. ^1^ https://tsh-receptor-mutation-database.org/ (accessed on 2 August 2023). ^2^ Data extracted from the website Varsome (https://varsome.com/, accessed on 2 August 2023). ^3^ Clustal Omega Multiple Sequence Alignment webtool (accessed on 2 August 2023, https://wwwdev.ebi.ac.uk/Tools/jdispatcher/msa/clustalo): +++ (0 changes); + (2 changes). ^4^ MutPred2: General MutPred score [0 (benign) − 1 (pathogenic)]; and Property score (0–1; probability to loss/gain the given property due to the sequence variant; the higher the score, the more likely that the molecular mechanism of the disease involves the alteration of the property). ^5^ Analyzed using MutPred-LOF (http://mutpred2.mutdb.org/mutpredlof/, accessed on 2 August 2023). ^6^ I-Mutant (https://folding.biofold.org/i-mutant/i-mutant2.0.html, accessed on 2 August 2023). ^7^ HGMD^®^ Professional 2023.2.

### 2.4. In Vitro Functional Studies

Our in vitro studies were based on the function of TSHR regarding its TSH-dependent activation of Gsα-coupled signal transduction. This signaling cascade activates the cAMP pathway, increasing the cAMP intracellular levels. We have analyzed these cAMP levels with a CRE luciferase reporter.

**Figure 3:**
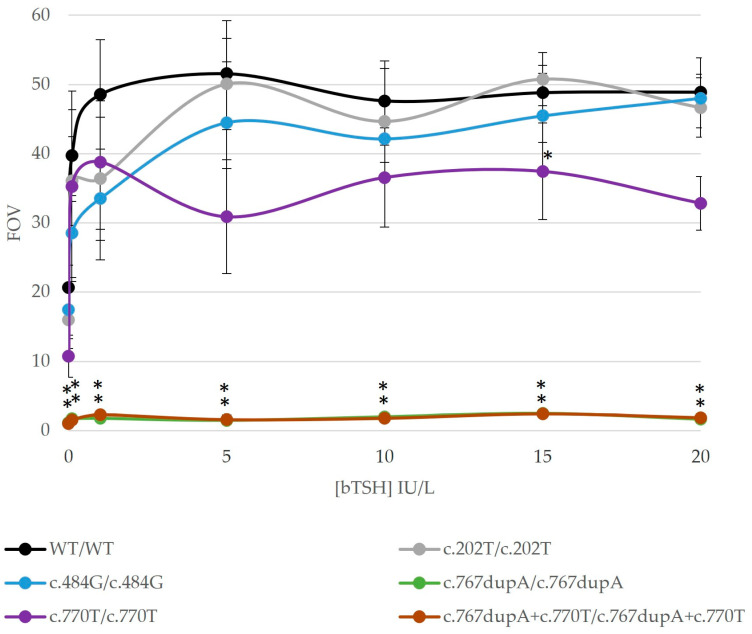
Results of in vitro functional studies of *TSHR* variants in our cohort. The TSH-dependent activation of Gsα-coupled signal transduction was studied in the detected variants in the homozygosis state. HEK293 cells were transiently transfected with a pGL4.29 (CRE) reporter and a TSHR-wild-type (WT) or a TSHR-mutant plasmid. The results of cells treated with different bovine TSH concentrations (0, 0.1, 1, 5, 10, 15 and 20 IU/L) are shown. Luciferase activity was measured with ONE-Glo™ Luciferase Assay System (Promega). Experiments were performed in duplicate. Results of the WT expression vector are shown in black and the ones from the mutant expression vectors in different colors. Data are shown in comparison with the empty vector values (FOV). The graphic shows impaired functional profiles in all the variants: completely deleterious [c.767dupA/p.(Asn256LysfsTer6)], two partially deleterious [c.484G/p.(Pro162Ala) and c.770T/p.(Thr257Ile)], and one mildly deleterious [c.202T/p.(Pro68Ser)]. Statistical significance was measured with a Student’s *t*-test (*p*-value < 0.05). Statistically significant differences with respect to WT are shown with *.

Phenotypes of CH-74 and CH-75 fitted with previous reports of their TSHR variants’ function (c.1657G>A and c.326G>A, respectively) and were not analyzed in vitro. We focused on the profiles in the homozygosis of the remaining TSHR mutants to determine the variant effect on patients CH-72, CH-74, and CH-77 (Figure 3). The *TSHR* variants in homozygosis showed variable functional profiles: one variant was completely deleterious (c.767dupA), two were partially deleterious (c.484G and c.770T), and one was mildly deleterious (c.202T) according to our results (Figure 2). Variants c.767dupA and c.770T were also analyzed together as they were in cis in our patient (CH-71), showing a completely deleterious profile in cis-homozygosis. Unfortunately, there were almost no statistically significant differences regarding mildly/partially deleterious variants due to high inter-experiment variability. Variant c.767dupA and a combination of variants c.767dupA/c.770T showed statistically significant differences versus WT with all TSH concentrations.

**Figure 4:**
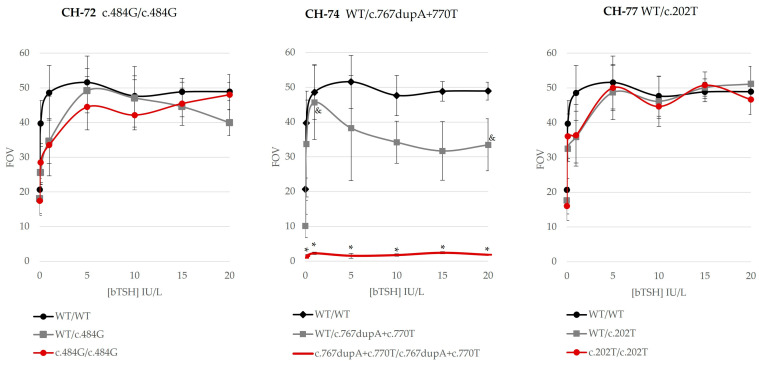
In vitro functional studies of our variants: studying the patients’ genotype in vitro. TSH-dependent activation of Gsα-coupled signal transduction was studied in all detected variants in homozygosis, heterozygosis, or cis-heterozygosis depending on the patient’s genotype. HEK293 cells were transiently transfected with a pGL4.29 (CRE) reporter and a TSHR-wild-type (WT), and/or a TSHR-mutant plasmid. The results of cells treated with different bovine TSH concentrations (0, 0.1, 1, 5, 10, 15, and 20 IU/L) are shown. Luciferase activity was measured with ONE-Glo™ Luciferase Assay System (Promega). Experiments were performed in duplicate. Results of the WT expression vector are shown in black, the ones of heterozygous mutant expression vectors in grey, and of homozygous mutant expression vectors in red. Data are shown in comparison with the empty vector values (FOV). Heterozygous variant c.202T/p.(Pro68Ser) in patient CH-77 showed mild impaired function. The homozygous c.484G/p.(Pro162Ala) variant in patient CH-72 showed partially impaired function. The two variants in the cis-heterozygosity of patient CH-71 showed partially impaired TSHR function, in spite of the presence of the completely deleterious c.767dupA/p.(Asn256LysfsTer6) variant. Statistical significance was measured with a one-way ANOVA test or a Kruskal–Wallis test depending on whether variances were equal or unequal, respectively (*p*-value < 0.05). Statistically significant differences: * variants versus WT are shown; & heterozygous versus homozygous variant.

Genotypes of the three patients were mimicked with our in vitro studies. Thus, the effect of the genotype of the patients was studied to determine the genotype–phenotype correlation (Figure 4). We analyzed both homozygous and heterozygous genotypes per variant and detected consistent genotype–phenotype correlation in our patients. Variant c.202T in heterozygosis in patient CH-77 (mild permanent CH, hypoplastic gland) showed mild impaired function. We also obtained a partial activity decrease in c.484G in homozygosis. CH-72, presenting this variant, was diagnosed with mild permanent CH. In addition, patient CH-71 (mild permanent CH), with two variants in the same allele (WT/c.767dupA+c.770T), showed partially impaired TSHR function, in spite of the presence of the completely deleterious c.767dupA variant.

All possible genotypes per variant were also compared, showing different outcomes depending on genotypes (Figure 4 and Figure S1). The four variants showed a different profile regarding the heterozygous or homozygous genotype. Variant c.202T presented a similar mildly deleterious profile in homozygosis and heterozygosis, whereas c.484G showed a partially deleterious profile with some differences. Regarding patient CH-77, we present all tested genotypes in vitro in Figure S1. Variant c.767dupA, completely deleterious in homozygosis (Figure 3 and Figure S1), showed a partial effect in heterozygosis (WT/c.767dupA) (Figure S1), whereas c.770T showed partial activity in homozygosis and heterozygosis (Figure S1). The two variants in cis-heterozygosis (WT/c.767dupA+c.770T) were partially deleterious (Figure 4), whereas, in cis-homozygosis (c.767dupA+c.770T/c.767dupA+c.770T), they were completely deleterious (patient CH-71, heterozygous) (Figure S1). Unfortunately, there were almost no statistically significant differences regarding partially deleterious variants due to high inter-experiment variability.

## 3. Discussion

We present five THD patients with eutopic glands carrying six *TSHR* variants. Two of these variants have not been previously reported: c.767dupA and c.770C>T. When the TSHR receptor function (signal transduction) is affected, this results in a decrease in T4 and T3 synthesis and secretion, with a compensatory increase in TSH secretion [6]. The degree of resistance to TSH may depend on the type and location of these variants. Thus, RTSH due to *TSHR* variants may give different phenotypes, depending on whether TSHR function is unaffected, partially affected, or completely affected: fully compensated RTSH (euthyroid state, appropriate increase in TSH); partially compensated RTSH (mild/borderline hypothyroidism); or uncompensated RTSH (severe CH) [8], respectively. Our patients, as previously described, presented partially compensated RTSH/mild hypothyroidism or uncompensated RTSH/severe CH and showed consistent genotype–functional–phenotype correlation, established thanks to in vitro and in silico functional studies.

Our in vitro studies were based on the TSH-dependent activation of the Gsα-coupled signal transduction of TSHR, which activates the cAMP pathway. The outcome of this pathway is the union of the cAMP response element-binding (CREB) transcription factor to a CRE sequence in a target gene promoter [17], with subsequent gene expression. Thus, we analyzed this pathway through a CRE luciferase reporter (CRE promoter coupled to the luciferase gene instead of a target gene). In summary, the TSHR mutants’ function was analyzed in different allelic dosages to check each *TSHR* variant effect (in homozygosis) and the genotypes according to our patients (genotype–phenotype study). In our system, TSHR mutants in homozygosis showed complete, partial, or mild misfunction (Figure 3). According to the literature, loss-of-function variants have been reported throughout the sequence, whereas gain-of-function variants are prevalent in the transmembrane domain but have usually been linked to hyperthyroidism [6,18].

The variants also showed different outcomes depending on their genotype state (Figure 4 and Figure S1): i.e., completely different in heterozygosity (partially deleterious) versus homozygosity (totally deleterious), for instance, variant c.767dupA; or showing a partial deficiency in both genotypes, like c.484G and c.770T. Finally, these results showed consistent genotype–phenotype correlation in our patients (Table 1, Figure 4).

Patient CH-71 presented two novel variants in the same allele (cis heterozygous): c.767dupA/p.(Asn256LysfsTer6) and c.770C>T/p.(Thr257Ile). Separately, variant c.767dupA was completely inactive, whereas c.770C>T was only partially inactive. Together, they presented full inactivation, as did variant c.767dupA alone (Figure 3, Figure 4 and Figure S1). The in vitro analysis mimicking the patient’s genotype (Figure 4) showed partially impaired TSHR activity of mutant TSHR-c.767dupA+c.770T. Thus, the WT receptor rescued the cis-heterozygous mutant. The patient had a mild permanent CH, which fits with partial TSHR function (Table 2). These results may also fit with the untreated hyperthyrotropinemia of his carrier father.

Variant c.202C>T/p.(Pro68Ser) showed a mild impaired function in a heterozygote state (Figure 4), which concurred with the mild permanent CH in patient CH-77. The patient also presented a hypoplastic gland and a low iodide uptake, which evolved to normal after reevaluation. Note that the father was a carrier; therefore, we may expect compensation from other factors. The variant was indeed previously reported in a homozygous patient with non-autoimmune subclinical hypothyroidism and her mother (with autoimmune thyroiditis) [11]. In this reported family, similar decreased cAMP levels with increasing doses of bovine TSH were found, but expression and affinity for TSH were lower than in WT [11]. This variant was also present in four heterozygous and two compound heterozygous patients with compensated resistance to TSH and showed a lower luciferase expression in this same study [8].

*TSHR* variant c.484C>G/p.(Pro162Ala) showed a partial decrease in activity in homozygosis and heterozygosis (Figure 3, Figure 4 and Figure S1); thus, it may be compatible with the mild CH in our patient CH-72. Our homozygous patient CH-72 was diagnosed with a mild permanent CH and had a low iodide uptake at reevaluation. His mother presented gestational CH. Similar to us, three previous studies showed partial activity and a partial membrane expression of this variant [7,14,15]. It was first detected in a family with three euthyroid siblings (high TSH levels, normal T4 levels) who were compound heterozygous p.(Pro162Ala)/p.(Asn167Ile) with their mother, who also carried c.484G [7]. Those assays showed partial function of the variant in both homozygosis and heterozygosis [7] and a reduced expression [15]. In addition, a study of hyperthyrotropinemic patients also showed a partial membrane expression and a partially affected signaling (Gs and Gq/11 pathways) of the variant [14].

Two patients presented two previously reported variants in homozygosity: [c.326G>A/p.(Arg109Gln)] in CH-75, and [c.1657G>A/p.(Ala553Thr)] in CH-74. In light of previous in vitro studies [12,16], we concluded that there was a clear genotype–phenotype correlation in our patients, and these variants were not studied in vitro. Variant c.326G>A (CH-75) was previously reported in a patient also carrying p.Trp546Ter: the patient showed TSH resistance, with highly increased serum TSH and low normal thyroid hormone levels [12]. This variant showed reduced TSH binding and an impaired signal transduction response to TSH [12]. Interestingly, it showed a dose–response shifted to the right and finally achieved the same activity as WT, at 10 IU/L. We have shown that this variant may also have an effect in heterozygosis, as the carrier father presents hyperthyrotropinemia. Previous studies have also shown that glutamine may protrude in the binding cavity and, therefore, the change to glutamine may interfere with TSH binding [12]. The second variant, c.1657G>A (CH-74), was previously reported in two siblings with very high TSH values and a very small thyroid gland, first mistaken for a thyroid agenesis [16]. The study of the variant in homozygosis showed extremely low expression at the cell surface but with an apparently normal synthesis inside the cell. The remaining membrane-expressed mutants bound to TSH with normal affinity and had normal cAMP production [16]. Similarly, Patient CH-74 (TSH = 100 mIU/L) had also a hypoplastic thyroid gland, and his reevaluation gammagraphy showed no iodine captation. Thus, our patient had a severe permanent CH, compatible with this extremely deleterious variant.

All the experiments were performed with the same conditions; however, unfortunately, there were not many statistically significant differences due to high inter-experiment variability. HEK293 cell line shows mismatch repair processes over time and the passage of cells may have been different for certain experiments. These facts may have contributed to this variability. Furthermore, bovine TSH was chosen over human recombinant TSH, as it showed to be more powerful than human in our pilot experiments. In addition, we have observed basal activity in the WT receptor and in all mutants. The basal (constitutive) activity is defined as the probability that part of the receptors expressed in the membrane adopt an active state without TSH binding [19]. Indeed, the constitutive activity of an unliganded TSH receptor related to the cAMP pathway has been reported [8,11,12,14,18], and some mutants have even shown high basal activity; yet, it is lower than the WT [8,11,14,18].

## 4. Patients, Materials, and Methods

### 4.1. Patients

The patients, from a DHT cohort of 160 patients belonging to the Catalan CH Neonatal Screening program, were treated at the Pediatric Endocrinology Section of the Hospital Universitari Vall d’Hebron. All patients, or their responsible parents or guardians, approved the use of their samples in research studies by informed consent. Their confidentiality was maintained by assigning a sample code. Our project was approved by the Clinical Research Ethics Committee (CEIC) of the Vall d’Hebron University Hospital (PR (AMI) 390/2016).

### 4.2. Inclusion Criteria, Biochemical Determinations, and Functional and Imaging Tests

The inclusion criteria were having TSH levels equal to or above 10 mIU/L at diagnostic confirmation of congenital hypothyroidism (CH), and gammagraphic and/or ultrasound studies suggestive of THD. We excluded patients with TSH levels under 10 mIU/L; or with imaging tests showing thyroid agenesis or ectopia; or syndromic patients. We also collected additional information on their relatives regarding thyroid disease or associated malformations/diseases.

Serum TSH levels were determined by electrochemiluminescence immunoassay (TSH3-Ultra, ADVIA Centaur^®^), and free T4 (fT4) levels were determined by electrochemiluminescence immunoassay (FT4, ADVIA Centaur^®^). Permanent or transient CH was determined using the results of thyroid function tests after temporary withdrawal of levothyroxine (LT4) therapy at approximately 3 years of age. After one month of the discontinuation of LT4 treatment, TSH and fT4 levels were measured in a peripheral blood sample. Individuals who showed continuous dependency on LT4 were diagnosed with permanent CH. After reevaluation, patients with TSH > 10 mIU/L (reference values 2–12 years: 0.6–4.1 mIU/L) and fT4 < 0.8 ng/dL (reference values 2–12 years: 0.8–1.4 ng/dL) were classified as severe permanent thyroid dyshormonogenesis (THD) and patients with TSH > 10 mIU/L and fT4 > 0.8 ng/dL as mild permanent THD. Therefore, these children were repeatedly evaluated at regular intervals of six months to monitor thyroid function. Those who did not need continuous LT4 therapy were diagnosed with transient CH (TSH < 5 mIU/L and fT4 > 0.8 ng/dL). Reevaluation was not carried out when LT4 requirements and genetic results were suggestive of THD.

### 4.3. Massive Sequencing and Genetic Variant Analysis

Pediatric patients with THD were analyzed using high-throughput techniques using a panel of 14 genes related to thyroid development and function: *ANO1*, *DUOX2*, *DUOXA2*, *FOXE1*, *GNAS*, *IYD*, *NKX2-1*, *NKX2-5*, *PAX8*, *TG*, *TPO*, *TSHR*, *SLC26A4*, and *SLC5A5*. DNA and amplicon preparation and massive sequencing were performed following the same protocols and using the same high throughput panel technology and sequencing platform as in Camats et al., 2021 [20].

Bioinformatic analysis of the obtained data was performed with MiSeq Control Software v2.6 (MCS), MiSeq Reporter (Illumina Inc, San Diego CA, USA), GeneRead SeqVariant Analysis software 1.10.0 (Qiagen, Hilden, Germany), and ANNOVAR [21] in order to evaluate the data quality, to align the sequences versus the human reference genome GRCh hg19, and to generate a list of variants of each patient. Variants with less than 20x coverage were not considered in the analysis. The classification of the variants was performed following the American College of Medical Genetics and Genomics (ACMG) criteria (accessed on 15 March 2021, https://www.acmg.net/) [10]. Prediction of the effect of variants identified in the splicing regions was evaluated using Alamut Visual 2.11 (accessed on 15 March 2021, https://www.interactive-biosoftware.com/es/alamut-visual/). Candidate variants, as well as target regions with low coverage (<20) were analyzed using Sanger sequencing. Family co-segregation was carried out, when possible. Variant nomenclature is according to the guidelines of the Human Genome Variation Society (HGVS) (accessed on 15 March 2021, https://www.hgvs.org/) [22].

### 4.4. In Silico Studies of TSHR Variants

All sequences were extracted from the NCBI database (accessed on 3 April 2023, https://www.ncbi.nlm.nih.gov). TSHR domains were constructed from the literature [6], the TSH Receptor Mutation Database (accessed on 1 September 2023, https://www.tsh-receptor-mutation-database.org/), and UniProt (AC P16473; accessed on 2 August 2023, https://www.uniprot.org/uniprotkb/P16473/entry). The sequence of human TSHR (thyrotropin receptor isoform 1 precursor, NP_000360.2) was aligned with TSHR peptide sequences of other species by the Clustal Omega Multiple Sequence Alignment (MSA) webtool (accessed on 2 August 2023, https://wwwdev.ebi.ac.uk/Tools/jdispatcher/msa/clustalo) [23]. MutPred2 webtool (accessed on 4 April 2023, http://mutpred2.mutdb.org/index.html#qform) was also used to predict the impact of variants on different protein properties [24]. I-mutant webtool (accessed on 4 April 2023, https://folding.biofold.org/i-mutant/i-mutant2.0.html) was used to predict protein sequence and protein structure stability. SpliceAI Webtool (accessed on 4 April 2023, https://spliceailookup.broadinstitute.org/) was used to predict any splicing defects. We also consulted the Varsome Database website (https://varsome.com/, accessed on 4 April 2023) and the Human Gene Mutation Database (HGMD) Professional (Biobase, Qiagen, Hilden, Germany).

### 4.5. In Vitro Functional Studies of TSHR Gene Variants

To determine the variant pathogenicity, in vitro functional studies were based on the TSH-dependent activation of the cAMP response element (CRE). This was measured using a CRE-reporter vector and a luciferase test.

#### 4.5.1. Plasmids and Cell Lines

An expression vector of wild-type TSHR was created with a TSHR cDNA from a control thyroid gland sample (amsbio Abingdon, UK) and a backbone of pcDNA3.1 empty vector (V790-20, Invitrogen, Thermo Fisher Scientific, Waltham, MA USA) using molecular cloning procedures. Mutant TSHR expression vectors corresponding to our *TSHR* variants c.202T, c.484G, c.767dupA, and c.770T were generated using PCR-based site-directed mutagenesis using specific primers and the QuickChange protocol (Agilent Technologies Inc., Santa Clara, CA, US). In addition, a reporter vector of the cAMP response element (CRE) for CREB (pGL4.29 luc2P/CRE/Hygro, E8471 vector, Promega, Madison, WI, USA) was purchased to detect the CRE-CREB union signal. Furthermore, HEK293 human cells (ECACC, UK, https://www.phe-culturecollections.org.uk/products/celllines, accessed on 4 April 2023) were cultured in DMEM (Gibco, Thermo Fisher Scientific), supplemented with 10% fetal calf serum, 1% penicillin/streptomycin, and 1% sodium pyruvate (Gibco, Thermo Fisher) in a 37 °C and 5% CO_2_ incubator.

#### 4.5.2. Cell Transfections, TSH Incubation, and Assay

For transient transfection and TSH-TSHR binding experiments, cells were trypsinized and plated in 96-well plates (lumox multiwell plates, ref. 94.6120.096, Sarstedt AG, Nümbrecht, Germany) at 35,000 cells/well. On the next day, the cells were transiently co-transfected with the pGL4.19 reporter construct (62.5 ng/well) and a TSHR-WT, a TSHR-mutant, or a TSHR-WT and a TSHR-mutant (12.5 ng/well TSHR-WT, 12.5 ng/well TSHR-mutant, or a TSHR-WT/TSHR-mutant 6.25 ng/well each construct) using Lipofectamine 2000TM (Invitrogen, Thermo Fisher). A pcDNA3.1 empty vector was used as a negative control. The medium was changed to a growth medium 6h after. Cells were monitored on the four days of the experiment.

For TSH-TSHR binding, a stock solution of bovine TSH (T8931, Sigma–Aldrich Solutions, Merck, Darmstadt, Germany) and inhibitor IBMX (Sigma–Aldrich Solutions, Merck, Darmstadt, Germany) were used, and subsequent dilutions were prepared to achieve adequate volumes to add to wells. On day four (48 h after transfection), the medium was replaced by fresh DMEM medium with or without different doses of bovine TSH per duplicate (0, 0.1, 1, 5, 10, 15, and 20 IU/L) and 0.5 mM IBMX with a total volume of 100 µL. Cells were incubated for 6h at 37 °C. One hour before the assay, cells were left at room temperature. A total of 100 µL of ONEGlo reactive (ONE-Glo™ Luciferase Assay System, Promega, Madison, US) was added to all the wells. Samples were immediately measured on a microplate luminescence reader (LUMIstar Omega, BMG Labtech GmbH, Ortenberg, Germany) with MARS Data Analysis Software v2 (BMG Labtech). Experiments were performed in duplicates and data were summarized and expressed, respecting the empty vector values (FOV, mean ± SEM). TSHR-WT and combinations of TSHR-WT/mutant or TSHR-mutant/mutant readings were compared. Statistical comparison was examined with a Student’s *t*-test, a one-way ANOVA test, or a non-parametric Kruskal–Wallis test with significance at *p* ≤ 0.05 using Microsoft Office Excel 2021 (Microsoft, Redmont, WA, USA) or SPSS software v20 (IBM Corp., Armonk, NY, USA).

## 5. Conclusions

In conclusion, 3.13% of our CH patients presented candidate variants in *TSHR*. These patients had THD with eutopic gland and carried six *TSHR* variants, with two of these having been not previously reported. Our patients, as previously described, presented mild or severe hypothyroidism and showed consistent genotype–functional–phenotype correlation, established thanks to in vitro and in silico functional studies. Our design of the in vitro functional studies has contributed to the confirmation of the pathogenicity of the variants studied. In addition, this approach highlights the importance of studying the effect of the patient’s genotype for a correct diagnostic confirmation.

## Figures and Tables

**Table 1:** Main biochemical and clinical characteristics and *TSHR* genetic findings.

THD Patient	Sex, YOB	conf TSH (mIU/L)/conf fT4 (ng/dL)	Tc-Gamm/US	Reevaluation (y)	I-Gamm/PDT	LT4 Dose (mcg/kg/day)	Final Diagnosis	Gene Change (NM_000369.2)	Zygosity	Cosegregation; Family History
CH-71	M, 2009	61.6/1.3	normal uptake/ND	Yes (4 y)	normal uptake/negative	1.8	Mild perm CH	c.767dupA/c.770C>T	cis-het	Fa/Fa; no
CH-72	M, 2013	33.2/1.1	low uptake/hypopl	No ^2^	ND/ND	4.03	Perm CH ^3^	c.484C>G	hom	Fa/Mo; Mo: gestational CH
CH-74	M, 2008	100/ND	ND/hypopl	Yes (4 y)	no uptake/negative	4.4	Severe perm CH	c.1657G>A	hom	ND; no
CH-75	M, 2017	71.7/1.1	low uptake/hypopl	No ^2^	ND/ND	3.8	Perm CH ^3^	c.326G>A	hom	Fa/Mo; Fa: hyperthyrotropinemia, untreated
CH-77	M, 2009	50 ^1^/1.14	low uptake/hypopl	Yes (3 y)	normal uptake/negative	2.28	Mild perm CH	c.202C>T	het	Fa; Fa: carrier, normal thyroid hormones, untreated

THD: thyroid dyshomonogenesis; YOB: year of birth; M: male; conf: at confirmation in the neonatal period; TSH: thyrotropin; fT4: free thyroxine; Tc-Gamm: gammagraphy with 99mTc (Technetium-99m); US: ultrasound; I-Gamm: gammagraphy with 123I at reevaluation; PDT: perchlorate discharge test; ND: not determined; hypopl: hypoplastic gland; y: years; hom: homozygous; cis-het: two heterozygous variants in cis; Fa: father; Mo: mother; perm: permanent. ^1^ screening value; ^2^ non-reevaluated due to elevated doses of LT4 treatment; ^3^ classifications by LT4 dose values.

## Data Availability

The original contributions presented in the study are included in the article/Supplementary Materials; further inquiries can be directed to the corresponding author.

## Supplementary Materials

The following supporting information can be downloaded at: https://www.mdpi.com/article/10.3390/ijms251810032/s1.
