# Supplementary material for: Genetic and Functional Studies of Patients with Thyroid Dyshormonogenesis and Defects in the TSH Receptor (TSHR)
Source: Int J Mol Sci. 2024 Sep 18;25(18):10032. doi: 10.3390/ijms251810032 (PMC11432690; doi:10.3390/ijms251810032)
Supplement: Supplementary file 1 [file ijms-25-10032-s001.zip › ijms-3210960-supplementary.pdf]

Genetic and functional studies of patients with thyroid dyshormonogenesis and defects in the TSH receptor (*TSHR*)

Diego Yeste, Noelia Baz-Redón, María Antolín, Elena Garcia-Arumí, Eduard Mogas, Ariadna Campos-Martorell, Núria Gonzalez-Llorens, Cristina Aguilar-Riera, Laura Soler-Colomer, María Clemente, Mónica Fernández-Cancio, Núria Camats-Tarruella.

SUPPLEMENTARY MATERIALS

Table S1. Clinical characteristics and anthropometric neonatal parameters of patients with *TSHR* variants.

Patient	Sex	GA (w)	Birth weight (g) (SD)	Birth lenght (cm) (SD)	Birth CP (cm) (SD)	Age first visit (d)
CH-71	M	40	4000 (+1.5)	51 (+0.4)	35 (0.0)	11
CH-72	M	39	4070 (+1.9)	52.5 (+1.5)	-	9
CH-74	M	39	2800 (-1.2)	46.5 (-2.0)	36 (+1.2)	7
CH-75	M	39	3100 (-0,5)	49.5 (-0,3)	35.5 (0.6)	7
CH-77	M	31	1110 (-2,0)	37 (-2,8)	27 (-0,7)	26

M: male; GA: gestational age; w: weeks; SD: standard deviation; CP: cephalic perimeter; d: days; -: not available.

Figure S1

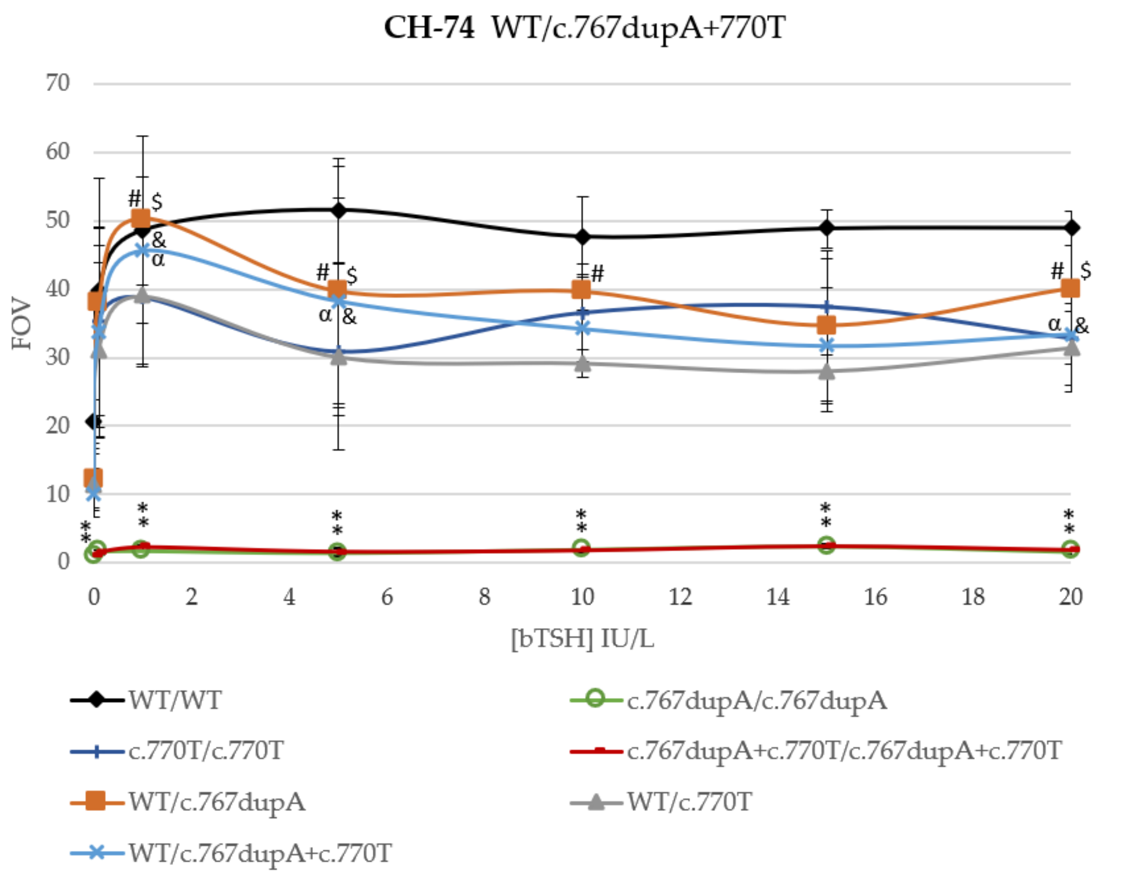

Figure S1. ***In vitro*** **functional studies of the described** ***TSHR*** **variants in patient CH-77: all tested genotypes.** TSH-dependent activation of Gsα-coupled signal transduction was studied in all detected variants in homozygosis, cis-homozygosis and cis-heterozygosis. HEK293 cells were transiently transfected with a pGL4.29 (CRE) reporter and a TSHR-wild-type (WT) and/or a TSHR-mutant plasmid, or TSHR-WT and TSHR-mutant plasmids. Cells were treated with different bovine TSH concentrations (0, 0.1, 1, 5, 10, 15 and 20 IU/L). Luciferase activity was measured with ONE-Glo™ Luciferase Assay System (Promega). Experiments were performed in duplicate. Data are shown in comparison with the empty vector values (FOV). The two homozygous genotypes including c.767dupA showed a totally deleterious effect, whereas the rest, those heterozygous or with the c.770T variant, showed a partially deleterious effect. Statistical significance was measured with a one-way ANOVA test or a Kruskal-Wallis test depending if variances were equal or unequal, respectively (p-value<0.05). Shown statistically significant differences: * mutants versus WT; **&** heterozygous WT/c.767dupA+c.770T versus homozygous c.767dupA+c.770T/c.767dupA+c.770T; **$** heterozygous wT/c.767dupA versus homozygous c.767dupA+c.770T; # heterozygous versus homozygous; **α** heterozygous WT/c.767dupA+c.770T versus homozygous c.767dupA/c.767dupA.
